# Relationship between sleep disorders and non-suicidal self-injury in adolescents with allergic rhinitis: a dual perspective analysis of person-centered and variable-centered on the role of social isolation

**DOI:** 10.3389/fpubh.2026.1795013

**Published:** 2026-05-11

**Authors:** Xiao Huang, Yi Wang, Shi Huang

**Affiliations:** Department of Otorhinolaryngology–Head and Neck Surgery, Clinical Medical College, The First Affiliated Hospital of Chengdu Medical College, Chengdu, China

**Keywords:** adolescents, allergic rhinitis patients, non-suicidal self-injury, sleep disorders, social isolation

## Abstract

**Background:**

Previous studies have established an association between sleep disorders and non-suicidal self-injury, yet the underlying psychological mechanisms of this relationship in adolescents with allergic rhinitis, a unique group with clear physical triggers and nighttime symptom burden, remain inadequately elucidated.

**Methods:**

This study employed a cross-sectional survey design and recruited allergic rhinitis adolescents aged 12 to 18 from five tertiary hospitals in Chengdu, China, between August and October 2025. Measurement and evaluation were conducted using the Sleep Disorders Screening Questionnaire, Social Isolation Scale, and Inventory of Statements About Self-injury.

**Results:**

Sleep disorders were positively correlated with social isolation (*r* = 0.475, *p* < 0.001) and NSSI (*r* = 0.385, *p* < 0.001), while social isolation was also positively correlated with NSSI (*r* = 0.405, *p* < 0.001). Mediation analysis showed that social isolation partially mediated the association between sleep disorders and NSSI, with an indirect effect of 0.154 [95% CI (0.104, 0.205)], accounting for 38.4% of the total effect. Latent profile analysis identified two subgroups: a low sleep disorder-low social isolation group (46.9%) and a high sleep disorder-high social isolation group (53.1%). Adolescents in the high sleep disorder-high social isolation group reported significantly higher NSSI scores than those in the low-risk group (*t* = −2.111, *p* = 0.035); this difference remained significant after adjustment for demographic and disease-related covariates (*F* = 7.454, *p* = 0.007).

**Conclusion:**

Through dual perspective analysis from individual-centered and variable-centered views, this study highlighted a significant association between sleep disorders and non-suicidal self-injury in adolescents with allergic rhinitis. In the clinical management of allergic rhinitis adolescents, routine screening and intervention for sleep disorders should be prioritized, alongside attention to the patients' social connectedness, and implementation of stratified, integrated preventive and intervention strategies for high-risk subgroups.

## 1 Introduction

Allergic rhinitis is an IgE-mediated chronic inflammatory disease (1), with a globally increasing prevalence, particularly prominent in the adolescent population (2, 3). Alnahas, Abouammoh (4) indicated that the global prevalence of allergic rhinitis in adolescents has reached 10 to 30%. The core clinical symptoms of this disease, including nasal congestion, itching, sneezing, and clear nasal discharge (5), not only impact the patients' daytime functioning but also significantly impair their nighttime sleep quality (6, 7). Of concern is that in the adolescent population, who are in a sensitive developmental phase, allergic rhinitis may exacerbate academic pressure, interpersonal barriers, and even self-harm behaviors (8, 9). However, existing studies have predominantly focused on the physiological mechanisms and pharmacological interventions of allergic rhinitis (10, 11), with insufficient discussion on the associated mental health issues, particularly the relationship between sleep disorders and non-suicidal self-injury. Therefore, this study aims to analyze the potential relationship and internal mechanisms between sleep disorders and non-suicidal self-injury in adolescents with allergic rhinitis, addressing a research gap and holding significant practical and theoretical value.

Sleep disorders are common comorbidities in allergic rhinitis patients (12, 13). This is primarily due to the nasal congestion, rhinorrhea, and nocturnal symptom fluctuations of allergic rhinitis directly disrupting sleep continuity (14, 15); additionally, the circadian rhythm changes in allergic inflammation and cytokine levels may influence sleep architecture through neuroimmune pathways (16, 17), leading to difficulties in falling asleep, increased nocturnal awakenings, and decreased sleep efficiency (18). Previous studies have shown that the prevalence of sleep disorders in adolescents with allergic rhinitis is 2 to 3 times higher than in healthy peers (19, 20), with an average reduction of 1–2 hours in sleep duration, accompanied by daytime sleepiness. For adolescents, chronic sleep deprivation or fragmented sleep not only impairs attention and executive function but also increases the risk of behavioral problems (21, 22). Specifically, chronic sleep deprivation can activate the stress response axis, increase difficulties in emotional regulation, and lead to abnormal behaviors in the adolescent population (23). Therefore, sleep disorders serve as a crucial link between physical disease burden and psychological behavioral issues.

Non-suicidal self-injury involves intentionally damaging one's body tissue without suicidal intent, often manifesting as cutting, burning, hitting, or scratching (24, 25). Previous research has found that non-suicidal self-injury is often utilized for emotional regulation (rapid relief of intense negative emotions or emptiness), self-punishment, gaining a sense of control, or signaling a need for help (26, 27); its occurrence is often associated with high emotional reactivity, low emotional regulation abilities, increased impulsivity, and inadequate social support (28). Sleep problems have been identified as a significant variable risk factor for non-suicidal self-injury (29). This is primarily because sleep deprivation weakens the prefrontal cortex's regulation of the limbic system, increasing emotional fluctuations and reducing behavioral inhibition (30, 31). Additionally, nightmares, nocturnal awakenings, and fatigue may increase the desire for pain tolerance, lower problem-solving abilities, and elevate the propensity for self-harm (32, 33). However, existing evidence largely comes from general adolescent populations or psychiatric samples, and whether the association between sleep disorders and non-suicidal self-injury is more pronounced in allergic adolescents with clear physical triggers and nighttime symptom burden, and the key psychological mechanisms involved, require further examination.

Social isolation is the negative emotional experience where individuals subjectively perceive a psychological distance or lack of connection with others, groups, or society, comprising components of loneliness, social exclusion, and lacking a sense of belonging (34, 35). For adolescents with allergic rhinitis, the recurrent symptoms of the disease may lead to decreased social participation, hindered peer interactions, and lowered self-image, thereby fostering intense feelings of social isolation (36). According to interpersonal psychological theories, feelings of belonging thwartedness and perceived burdensomeness are core psychological factors driving self-harm and suicidal behaviors, with social isolation serving as a significant indicator of belongingness deprivation (37). Specifically, the fatigue, attention deficits, and emotionally irritability caused by sleep disorders may further deteriorate the quality of peer interactions and parent-child communications, accumulating into stronger experiences of isolation (38, 39). From the perspective of belongingness need theory (40), feelings of isolation can act as a psychological manifestation of chronic stress, enhancing feelings of helplessness, loneliness, and negative self-evaluation (41); on the other hand, it may weaken the protective effects of social support, leading individuals to adopt non-suicidal self-injury as a short-term, private, and highly reinforcing coping mechanism (42).

While existing studies have preliminarily established the association between sleep disorders and social isolation (43), specific evidence for the adolescent population with allergic rhinitis remains scarce, especially lacking mediation analysis considering social isolation. Although traditional variable-centered methods can provide an overall trend, they often mask subgroup differences (44, 45), for instance, adolescents with high sleep disorders-high social isolation may exhibit stronger non-suicidal self-injury tendencies, unlike those with low sleep disorders-low social isolation. The individual-centered approach addresses this limitation by identifying latent categories, revealing heterogeneous mechanisms, and providing the basis for precise interventions (46). By utilizing a combined dual-perspective approach, this study aims to enhance the robustness of research and avoid biases associated with a single method.

Focusing on adolescents with allergic rhinitis, this study systematically examines the relationship between sleep disorders and non-suicidal self-injury from both variable-centered and individual-centered perspectives, considering the mechanism of social isolation. Specifically, we first investigate the association between sleep disorders and non-suicidal self-injury within the variable-centered framework and examine the mediating role of social isolation to clarify whether the mediation path “sleep disorders-social isolation-non-suicidal self-injury” holds; secondly, under the individual-centered framework, we identify potential joint profiles of sleep disorders and social isolation, testing the heterogeneous structure of sleep disorders and isolation experiences in adolescents with allergic rhinitis; finally, we compare different profiles' buffering effects on non-suicidal self-injury. Through this dual-perspective design, the study aims to provide a mechanism-based and population-specific evidence base for early identification and stratified interventions for non-suicidal self-injury in adolescents with allergic rhinitis, and offer testable theoretical support for integrating sleep and social connection interventions into the management of otolaryngology chronic diseases.

Based on the above analysis, this study proposes the following hypotheses:

**H1:** Sleep disorders are positively associated with non-suicidal self-injury in adolescents with allergic rhinitis.
**H2:** Social isolation statistically mediates the association between sleep disorders and non-suicidal self-injury.
**H3:** Adolescents with allergic rhinitis exhibit significant heterogeneity in joint profiles of sleep disorders and social isolation.

## 2 Method

### 2.1 Research design

The study utilized a cross-sectional survey design to investigate the relationship between sleep disorders and non-suicidal self-injury in adolescents with allergic rhinitis. Given the sensitive nature of the psychological variable of non-suicidal self-injury in this study, the cross-sectional design effectively collected the necessary data while safeguarding participant privacy and laying the groundwork for future longitudinal tracking studies. Data collection took place from August to October 2025.

### 2.2 Participant recruitment

#### 2.2.1 Recruitment process

This study used a multicenter hospital-based consecutive sampling strategy rather than strict probability random sampling. Specifically, five tertiary hospitals in Chengdu, China, including three general tertiary hospitals and two pediatric specialty hospitals, were selected as study sites based on feasibility, case accessibility, and institutional collaboration. Within each participating hospital, adolescents with allergic rhinitis who presented to the otolaryngology clinics or wards during the study period and met the eligibility criteria were consecutively approached for recruitment.

Potential participants and their legal guardians were given detailed information regarding the study purpose, procedures, confidentiality protection, and potential risks, and written informed consent was obtained from both adolescents and guardians before participation. Eligible participants then completed the electronic questionnaires in a private and quiet setting under researcher guidance. Questionnaire completeness was checked on site, and missing responses were completed only with the participant's consent.

#### 2.2.2 Inclusion and exclusion criteria

##### 2.2.2.1 Inclusion criteria

Participants were eligible if they met all of the following criteria: (1) Age between 12 and 18 years; (2) Confirmed diagnosis of allergic rhinitis made by an otolaryngologist, based on typical clinical symptoms and either a positive skin prick test or positive serum allergen-specific IgE results; (3) Disease duration ≥2 months, to ensure that the potential psychosocial impact of a chronic condition could be adequately perceived; (4) Basic Chinese literacy, enabling independent understanding and completion of the questionnaire; (5) Written informed consent provided by both the adolescent and their legal guardian.

##### 2.2.2.2 Exclusion criteria

Participants were excluded if they met any of the following conditions: (1) Presence of other chronic respiratory diseases (e.g., bronchial asthma, COPD, chronic rhinosinusitis) to reduce confounding effects of respiratory comorbidity on sleep disturbance measurement; (2) A history of, or current, major psychiatric disorders diagnosed by a psychiatrist (e.g., schizophrenia, bipolar disorder, major depressive disorder); (3) Severe cognitive impairment; (4) Use of medications within 2 weeks prior to data collection that may affect sleep architecture, including sedative-hypnotics, antihistamines, and intranasal corticosteroids. This criterion was introduced to reduce pharmacological confounding in the assessment of sleep-related variables; however, we acknowledge that it may also limit the representativeness of the sample, particularly because antihistamines and intranasal corticosteroids are commonly used in routine allergic rhinitis management.

#### 2.2.3 Minimum sample size

For variable-centered analysis, the study calculated the sample size using G^*^Power 3.1 software, setting the effect size at a medium level (f^2^ = 0.15), significance level α at 0.05, statistical power (1-β) at 0.95, and predicting eight variables. The minimum sample size required for structural equation model analysis was determined to be 160 cases. Following Nylund-Gibson and Choi (47) recommendations, the sample size for latent profile analysis should ensure each profile comprises at least 5 to 10% of the total sample to ensure stable and reliable parameter estimation. Assuming three to five potential profiles might be identified in the study, the total sample size was estimated to be no less than 300 cases.

#### 2.2.4 Study sample

During the data collection period, 612 potential participants were approached, with 487 meeting all inclusion criteria and not meeting any exclusion criteria, resulting in an initial inclusion rate of 79.58%. Among the 125 potential participants who were not included, 47 were excluded due to comorbid asthma or chronic sinusitis, 38 due to a disease duration of less than 2 months, 12 due to a history of significant psychiatric disorders, and 28 due to refusal to sign informed consent forms. Following data quality checks, 14 invalid questionnaires were excluded, resulting in a final sample of 473 participants for statistical analysis, with an effective recovery rate of 97.13%, exceeding the minimum target sample size and meeting the requirements for subsequent statistical analyses.

### 2.3 Ethical considerations

The study adhered to the Helsinki Declaration and the ethical standards of the institution, receiving approval from the hospital/university ethics committee before implementation (No.: 2025CYFYIRB-BA-156). Given the involvement of minors and sensitive information on non-suicidal self-injury, informed consent was obtained both from legal guardians and adolescents, emphasizing anonymity and confidentiality to enhance data privacy and security.

### 2.4 Study tools

#### 2.4.1 Sleep disorders scale

Sleep disorder was assessed using the Sleep Disorders Scale–Brief developed by Yu, Buysse (48), which comprises 8 items. The scale was translated using a back-translation procedure in the study by Yu, Zhu (49) (translated by two Chinese psychology students) and demonstrated satisfactory cultural adaptation and reliability in Chinese samples. Participants rated their sleep disorder across three domains: sleep satisfaction, sleep quality, and sleep interference. Items were scored on a 5-point Likert scale (1 = not at all, 5 = very much), yielding a total score ranging from 8 to 40, with higher scores indicating more severe sleep disorder. In the present study, the scale showed excellent internal consistency (Cronbach's α = 0.903). A single-factor measurement model was specified in AMOS 30.0; confirmatory factor analysis (CFA) indicated good model fit, as shown in Table 1.

**Table 1:** Indicators of model fit for the study variables.

Variables	χ^2^/df	GFI	AGFI	RMSEA	CFI	TLI	Factor loading
Sleep disorder	1.279	0.986	0.976	0.024	0.996	0.995	0.587~0.748
Social isolation	1.099	0.974	0.965	0.015	0.998	0.997	0.616~0.791
Non-suicidal self-injury	1.001	0.982	0.974	0.010	0.996	0.997	0.631~0.783

χ^2^/df, chi-square to degrees of freedom ratio; GFI, goodness-of-fit index; AGFI, adjusted goodness-of-fit index; RMSEA, root mean square error of approximation; CFI, comparative fit index; TLI, Tucker-Lewis index.

Acceptable model fit criteria: χ^2^/df < 3, GFI > 0.90, AGFI > 0.90, RMSEA < 0.08, CFI > 0.90, TLI > 0.90.

#### 2.4.2 Social isolation scale

Social isolation was measured using the Social Isolation Scale developed by Jessor and Jessor (50), consisting of 15 items. The scale was translated into Chinese by Wu, Li (51), who also supported its cultural suitability and reliability in an adult sample. In this study, the scale was used to assess perceived social isolation among adolescents with allergic rhinitis. Responses were rated on a 5-point Likert scale (1 = strongly disagree to 5 = strongly agree). Total scores range from 15 to 75, with higher scores indicating greater social isolation. Internal consistency in the current sample was excellent (Cronbach's α = 0.938). A single-factor model was tested in AMOS 30.0, and CFA results demonstrated good model fit (see Table 1).

#### 2.4.3 Non-suicidal self-injury scale

The non-suicidal self-injury was assessed using the Inventory of Statements About Self-Injury (ISAS) developed by Klonsky and Glenn (52), which includes 12 items. The Chinese version was produced by Yu, Zhu (49) using back-translation and was validated for cultural applicability and reliability in Chinese adolescent populations. The instrument assesses 12 types of deliberate self-injurious behaviors without suicidal intent, such as hitting oneself, biting, burning, and carving, among others. Items were rated on a 5-point Likert scale ranging from 1 (never) to 5 (five times or more). Total scores range from 12 to 60, with higher scores indicating greater non-suicidal self-injury engagement. In the present study, internal consistency was excellent (Cronbach's α = 0.925). A single-factor model was specified in AMOS 30.0, and CFA results indicated good model fit (see Table 1).

### 2.5 Statistical analysis

The study employed SPSS 27.0, AMOS 30.0, and Mplus 8.3 software for data management and statistical analysis with a significance level (α) set at 0.05. Internal consistency reliability and confirmatory factor analysis were used to assess the scales' reliability and structural validity. Internal consistency was evaluated using Cronbach's α coefficient (≥0.7 as an acceptable standard). Confirmatory factor analysis in AMOS 30.0 software utilized maximum likelihood estimation for parameter estimation.

Firstly, Harman's single-factor test was used to measure common method bias, with a threshold set at 40%. Secondly, descriptive statistics were employed to analyze variable distribution characteristics and central tendencies, presented as mean ± standard deviation. Thirdly, independent sample *t*-tests or one-way analysis of variance were used to examine intergroup differences in non-suicidal self-injury, with effect sizes quantified using Cohen's *d* or η^2^. Fourthly, correlation analysis was conducted to test the linear relationship between continuous variables. Fifthly, the mediation model of social isolation was examined using Model 4 in the SPSS PROCESS macro. Statistical inference for the mediation effect utilized bias-corrected percentile bootstrap method with 5,000 resamples, and a significant indirect effect was determined if the 95% confidence interval did not include zero.

Sixthly, latent profile analysis from an individual-centered perspective was performed using Mplus 8.3 software, with sleep disorders and social isolation scores used as continuous indicator variables to identify heterogeneous subtypes in the adolescent population. The determination of the optimal number of profiles involved fitting models from a single-profile model to a five-profile model. Finally, independent sample *t*-tests were used to compare differences in non-suicidal self-injury scores among different latent profiles.

## 3 Results

### 3.1 Demographic characteristics

A total of 473 eligible adolescents with allergic rhinitis were included. The mean age was 14.98 years (*SD* = 2.026). Among them, 265 were male (56.00%) and 208 were female (44.00%). Most participants were in junior high school (*n* = 244, 51.60%), and 275 resided in urban areas (58.10%). Time since diagnosis was predominantly ≥ 1 year (*n* = 130, 27.50%), and 181 participants (38.30%) reported symptom episodes occurring 3–4 times per week. Detailed demographic characteristics are presented in Table 2.

**Table 2:** Demographic information of participants.

Variable	Items	Number (*N*)	Frequency (%)
Gender	Male	265	56.00%
	Female	208	44.00%
Educational background	Primary school and below	129	27.30%
	Junior high school	244	51.60%
	Senior high school	100	21.10%
Place of residence	Urban	275	58.10%
	Rural areas	198	41.90%
Time of diagnosis	2–3 months	116	24.50%
	3–6 months	119	25.20%
	6–12 months	108	22.80%
	1 years and above	130	27.50%
Number of episodes per week^*^	< 3 times	139	29.40%
	3–4 times	181	38.30%
	5 times and above	153	32.30%
Age	*M* ±*SD*	14.98 ± 2.026	

M, mean; SD, standard deviation.

^*^Number of episodes per week refers to the average frequency of rhinitis symptoms (e.g., sneezing, runny nose, nasal congestion, itching) experienced per week during the past 4 weeks or the most recent allergy season.

### 3.2 Assessment of common method bias

To evaluate the potential impact of common method bias, we conducted Harman's single-factor test. All 35 measurement items were entered into an unrotated exploratory factor analysis. Three factors with eigenvalues >1 were extracted, and the first factor accounted for 34.265% of the total variance, which is below the conventional 40% threshold. These findings suggest that common method bias was not a serious concern in this study.

### 3.3 Descriptive statistics and correlation analysis

Descriptive statistics for the study variables are shown in Table 3. According to the criteria proposed by Kline (53), skewness values for the core variables ranged from −0.111 to −0.020, and kurtosis values ranged from −0.294 to −0.477, indicating that the distributions were broadly consistent with normality assumptions.

**Table 3:** Descriptive statistics and correlations of the study variables.

Variables	*M*	*SD*	Skewness	Kurtosis	1	2	3
Sleep disorders	2.984	0.726	−0.020	−0.377	1		
Social isolation	3.018	0.739	−0.111	−0.477	0.475^***^	1	
Non-suicidal self-injury	3.306	0.740	−0.081	−0.294	0.385^***^	0.405^***^	1

^***^p < 0.001.

As shown in Table 3, sleep disorders was positively correlated with social isolation (*r* = 0.475, *p* < 0.001) and with non-suicidal self-injury (*r* = 0.385, *p* < 0.001). social isolation was also positively correlated with non-suicidal self-injury (*r* = 0.405, *p* < 0.001). All correlation coefficients fell between 0.30 and 0.50, reflecting moderate associations, and the observed pattern was consistent with theoretical expectations. These effect sizes suggest that sleep disorder, social isolation, and non-suicidal self-injury are meaningfully related. However, the correlations were not so large as to indicate that any single factor alone is sufficient for clinical identification, underscoring the need for multidimensional assessment in adolescents with allergic rhinitis.

### 3.4 Group comparisons

To examine differences in non-suicidal self-injury scores across demographic and disease-related characteristics, independent-samples *t* tests or one-way ANOVAs were conducted as appropriate (Table 4).

**Table 4:** Group differences in demographic information regarding non-suicidal self-injury.

Variable	Items	*M*	*SD*	*t*/*F*	*p*	Cohen's *d*/η^2^
Gender	Male	3.210	0.762	−3.205	0.001	−0.297
	Female	3.428	0.693			
Educational background	Primary school and below	3.534	0.745	13.043	< 0.001	0.053
	Junior high school	3.293	0.701			
	Senior high school	3.044	0.741			
Place of residence	Urban	3.285	0.770	−0.744	0.457	−0.069
	Rural areas	3.336	0.696			
Time of diagnosis	2–3 months	3.102	0.709	5.581	< 0.001	0.034
	3–6 months	3.277	0.747			
	6–12 months	3.353	0.698			
	1 years and above	3.476	0.756			
Number of episodes per week	< 3 times	3.234	0.760	1.089	0.337	0.005
	3–4 times	3.357	0.728			
	5 times and above	3.310	0.734			

Cohen's d was used as the effect size measure for independent samples t-tests; η^2^ (eta-squared) was used as the effect size measure for one-way ANOVA.

Female participants reported significantly higher non-suicidal self-injury scores (*M* = 3.428, *SD* = 0.693) than male participants (*M* = 3.210, *SD* = 0.762), and the difference was statistically significant (*t* = −3.205, *p* = 0.001, Cohen's *d* = −0.297). This finding indicates a meaningful gender difference, suggesting that female adolescents with AR may represent a higher-risk subgroup for non-suicidal self-injury.

One-way ANOVA indicated significant differences in non-suicidal self-injury across educational levels (*F* = 13.043, *p* < 0.001, η^2^ = 0.053). Specifically, non-suicidal self-injury scores were highest among those with primary school education or below (*M* = 3.534, *SD* = 0.745), followed by the junior high school group (*M* = 3.293, *SD* = 0.701), and lowest in the high school group (*M* = 3.044, *SD* = 0.741). This suggests a decreasing trend in non-suicidal self-injury with higher educational attainment, implying that younger participants with lower educational levels may face greater risk.

Non-suicidal self-injury also differed significantly by time since diagnosis (*F* = 5.581, *p* < 0.001, η^2^ = 0.034). Participants with a diagnosis duration of ≥ 1 year reported higher non-suicidal self-injury scores (*M* = 3.476, *SD* = 0.756) than those in other diagnostic-duration groups. This pattern suggests that longer-term disease burden may contribute to elevated non-suicidal self-injury risk among adolescents with AR.

In contrast, there was no significant difference in non-suicidal self-injury between urban (*M* = 3.285, *SD* = 0.770) and rural participants (*M* = 3.336, *SD* = 0.696) (*t* = −0.744, *p* = 0.457, Cohen's *d* = −0.069). Likewise, non-suicidal self-injury did not differ significantly across symptom-frequency groups (*F* = 1.089, *p* = 0.337, η^2^ = 0.005). These findings suggest that residence and symptom episode frequency were not major correlates of non-suicidal self-injury in this sample.

### 3.5 Mediation analysis of social isolation

To test whether social isolation mediated the association between sleep disorders and non-suicidal self-injury, we conducted a mediation analysis using PROCESS v4.1 (Model 4) in SPSS. Sleep disorders was specified as the independent variable, non-suicidal self-injury as the dependent variable, and social isolation as the mediator, while age, gender, educational level, place of residence, number of symptom, and time since diagnosis were included as covariates.

**Figure 1:**
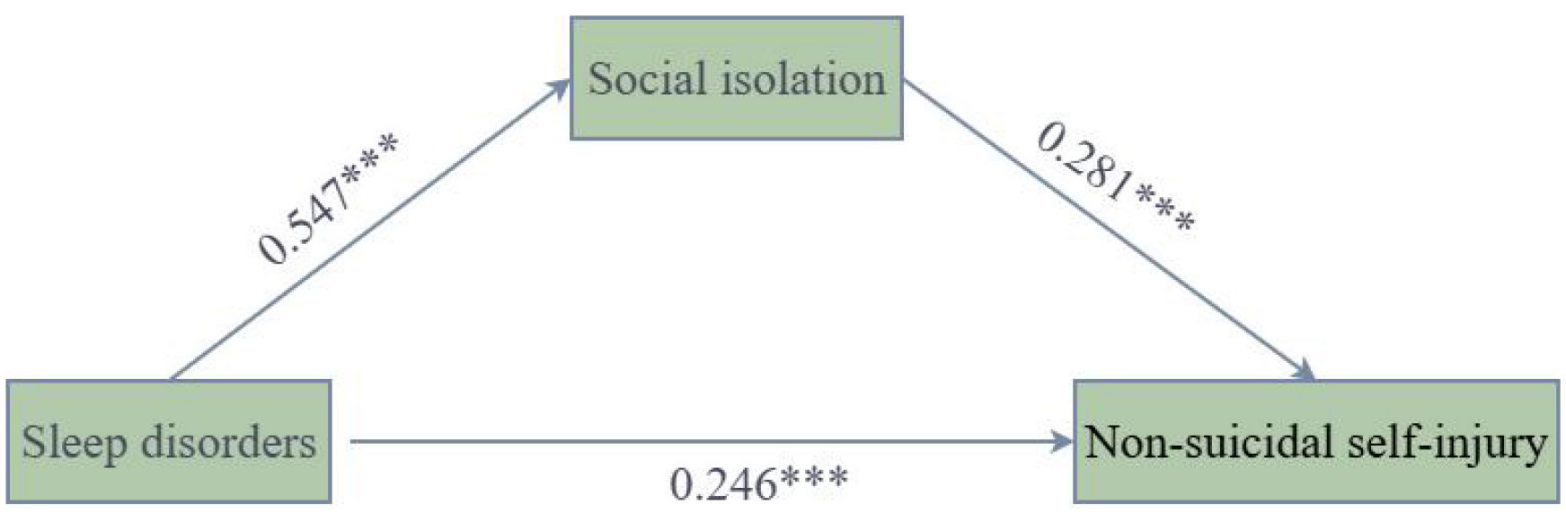
Path coefficients of the mediating effect of social isolation. ***p < 0.001.

Regression results are presented in Table 5 and Figure 1. In Model 1, with social isolation as the outcome, sleep disorders were significantly and positively associated with social isolation after controlling for covariates (*B* = 0.547, *p* < 0.001, 95% CI [0.449, 0.646]), indicating that adolescents with more severe sleep disorders reported stronger social isolation. In Model 2, with non-suicidal self-injury as the outcome, when both sleep disorders and social isolation were entered simultaneously (and the same covariates were controlled), the direct effect of sleep disorders on non-suicidal self-injury remained significant (*B* = 0.246, *p* < 0.001, 95% CI [0.139, 0.353]), and social isolation was also significantly associated with non-suicidal self-injury [*B* = 0.281, *p* < 0.001, 95% CI (0.193, 0.368)]. These results indicate that sleep disorders retained a significant association with non-suicidal self-injury even after accounting for social isolation.

**Table 5:** Regression analysis results of the mediating effect of social isolation.

Model	Predictor	Outcome	Model fitting		Regression coefficient index			
			*R* ^2^	*F*	*B*	*t*	LLCI	ULCI
Model 1	Sleep disorders	Social isolation	0.245	21.533^***^	0.547	10.906	0.449	0.646
	Age				−0.003	−0.184	−0.032	0.026
	Educational level				0.022	0.474	−0.069	0.112
	Place of residence				−0.010	−0.163	−0.129	0.109
	Time since diagnosis				0.011	0.403	−0.041	0.063
	Number of symptom				0.093	2.427	0.018	0.169
	Gender				−0.158	−2.043	−0.311	−0.006
Model 2	Sleep disorders	Non-suicidal self-injury	0.300	24.857^***^	0.246	4.535	0.139	0.353
	Social isolation				0.281	6.275	0.193	0.368
	Age				0.019	1.299	−0.010	0.047
	Educational level				−0.262	−5.908	−0.350	−0.175
	Place of residence				0.090	1.535	−0.025	0.205
	Time since diagnosis				0.098	3.851	0.048	0.148
	Number of symptom				0.001	0.023	−0.073	0.074
	Gender				0.055	0.734	0.092	0.203

^***^p < 0.001.

CI, confidence interval

R^2^, coefficient of determination, indicating the proportion of variance explained by the model;

F, F-statistic for overall model significance;

β, standardized regression coefficient;

t, t-statistic for individual predictor significance;

CI, confidence interval.

Bootstrap results are shown in Table 6. The total effect of sleep disorders on non-suicidal self-injury was 0.400 (*SE* = 0.050, 95% CI [0.301, 0.499]), indicating a significant overall association. After inclusion of social isolation, the direct effect of sleep disorders on non-suicidal self-injury remained significant [β = 0.246, *SE* = 0.054, 95% CI (0.139, 0.353)]. The indirect effect via social isolation was 0.154 [*SE* = 0.026, 95% CI (0.104, 0.205)], supporting a significant partial mediation pathway: sleep disorders → social isolation → non-suicidal self-injury. In other words, sleep disorders were associated with higher non-suicidal self-injury, both directly and indirectly in the statistical model through higher perceived social isolation.

**Table 6:** Bootstrap test results for mediation effects.

Type of effect	β	*SE*	LLCI	ULCI	Effect proportion	Supporting hypothesis
Total effect	0.400	0.050	0.301	0.499	100.00%	
Direct effect	0.246	0.054	0.139	0.353	61.6%	H1
Indirect effect	0.154	0.026	0.104	0.205	38.4%	H2

SE, standard error; LLCI, lower limit of 95% confidence interval; ULCI, upper limit of 95% confidence interval.

Indirect effect represents the mediation pathway: sleep disorders → social isolation → non-suicidal self-injury.

In terms of practical magnitude, the indirect pathway through social isolation accounted for 38.99% of the total effect, indicating that more than one-third of the association between sleep disorder and non-suicidal self-injury may operate through perceived social disconnection. This proportion suggests that social isolation is not merely a statistically significant mediator but also a clinically meaningful intervention target. In addition, the final model explained 29.4% of the variance in non-suicidal self-injury, which indicates a moderate level of explanatory value for psychosocial research in this population.

### 3.6 Joint latent profile analysis of sleep disorders and social isolation

Latent profile analysis was conducted in Mplus 8.3, using the 8 sleep disorders items and 15 social isolation items as continuous indicators. Models ranging from one to five profiles were estimated, and fit indices are presented in Table 7.

**Table 7:** Comparison of fit indices of latent profile models for profile 1 to 5.

Profile	AIC	BIC	aBIC	Entropy	LMR (*P*)	BLRT (*P*)	Proportion of potential subgroups
1	31,007.755	31,199.073	31,053.077	—	—	—	—
2	27,926.360	28,217.496	27,995.328	0.935	< 0.001	< 0.001	0.469/0.531
3	27,225.593	27,616.548	27,318.207	0.895	0.713	< 0.001	0.249/0.402/0.348
4	26,895.063	27,385.837	27,011.324	0.887	0.401	< 0.001	0.163/0.268/0.292/0.277
5	26,573.580	27,164.172	26,713.487	0.897	0.219	< 0.001	0.152/0.280/0.186/0.220/0.162

AIC, Akaike information criterion; BIC, Bayesian information criterion; aBIC, sample-size adjusted Bayesian information criterion; LMR, Lo-Mendell-Rubin likelihood ratio test; BLRT, bootstrap likelihood ratio test.

As the number of profiles increased, AIC, BIC, and adjusted BIC decreased monotonically, suggesting incremental improvements in model fit. For the LMR-LRT, the two-profile solution fit significantly better than the one-profile model (*p* < 0.001). However, the LMR-LRT was non-significant for the three-profile (*p* = 0.713), four-profile (*p* = 0.401), and five-profile (*p* = 0.219) solutions, indicating that adding additional profiles did not yield statistically meaningful improvement. The BLRT was significant for all models (*p* < 0.001), suggesting improved fit with additional profiles from a purely statistical standpoint. The two-profile solution yielded the highest entropy (0.935), indicating clearer classification than alternative solutions. Moreover, class proportions were 46.9 and 53.1%, respectively, suggesting stable subgroup sizes. Considering the LMR-LRT, entropy, and parsimony, the two-profile model was selected as the optimal solution, supporting H3.

**Figure 2:**
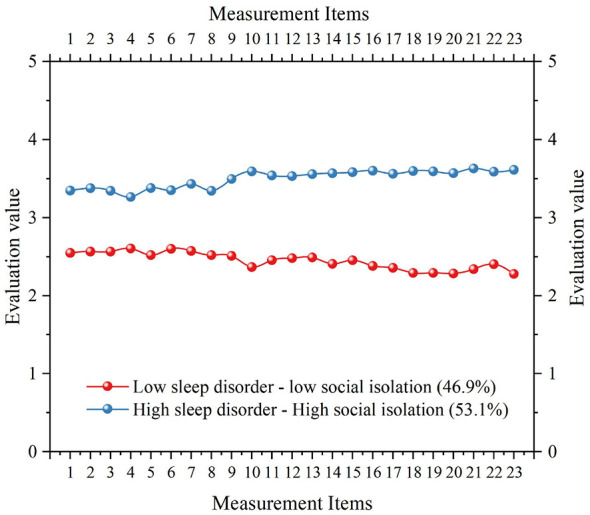
Two latent profiles in sleep disorder with social isolation in each index score pattern.

Based on the two-profile solution, profile plots were generated in Origin 2025 (Figure 2). The “low sleep disorder–low social isolation” profile (46.9%) reflected relatively good sleep, with fewer symptoms such as sleep initiation difficulties, nocturnal awakenings, and daytime fatigue, alongside stronger psychological connectedness with peers, family, and society (greater perceived belonging and social support). This profile may be conceptualized as a low-risk adaptive group among adolescents with AR. The “high sleep disorder–high social isolation” profile (53.1%) was characterized by pronounced sleep initiation and maintenance problems, impaired daytime functioning, and stronger perceived psychological distance and disconnection in peer relationships, family interactions, and social participation. In this subgroup, sleep disorder and social isolation appeared to co-occur and accumulate, consistent with a high-risk vulnerable group.

### 3.7 Differences in non-suicidal self-injury across latent profiles

To examine whether non-suicidal self-injury differed by latent profile membership, an independent-samples *t*-test was conducted (Table 8). The high sleep disorder–high social isolation group reported significantly higher non-suicidal self-injury scores (*M* = 3.373, *SD* = 0.695) than the low sleep disorder–low social isolation group (*M* = 3.229, *SD* = 0.783), with a statistically significant difference (*t* = −2.111, *p* = 0.035). This finding indicates that the joint high-risk profile of elevated sleep disorder and social isolation is associated with greater non-suicidal self-injury risk relative to the low-risk adaptive profile. However, the effect size was small (Cohen's *d* = −0.195), suggesting that the between-profile difference may be more informative for population-level risk stratification and early screening than for strong individual-level clinical discrimination.

**Table 8:** Comparison of differences in non-suicidal self-injury among different latent profiles.

Latent profiles	*M*	*SD*	*t*	*p*	Cohen's *d*
Low sleep disorder-low social isolation (46.9%)	3.229	0.783	−2.111	0.035	−0.195
High sleep disorder-high social isolation (53.1%)	3.373	0.695			

To further exclude the confounding effects of demographic and disease-related factors, we performed an analysis of covariance (ANCOVA) with age, gender, educational level, place of residence, number of symptom, and time since diagnosis as covariates. The results showed that after controlling for these covariates, the effect of latent category on NSSI remained significant (*F* = 7.454, *p* = 0.007), indicating that the high sleep disorder-high social isolation group still exhibited higher levels of NSSI.

## 4 Discussion

### 4.1 Variable-centered findings

Under a variable-centered framework, this study confirmed a significant positive association between sleep disorder and non-suicidal self-injury, consistent with prior findings in general adolescent and psychiatric samples (54, 55). The distinctive contribution of the present work is that this association was examined within the specific context of adolescents with allergic rhinitis. In allergic rhinitis, nocturnal nasal obstruction, night-to-night symptom fluctuation, and circadian variation in inflammatory mediators commonly impair sleep quality (56). Such sleep problems, driven by identifiable physiological triggers, may be more chronic and persistent, thereby exerting a deeper and more sustained impact on adolescents' emotional regulation. From a neurobiological perspective, sleep deprivation can weaken top–down prefrontal control over limbic structures such as the amygdala, increasing reactivity to negative stimuli while reducing regulatory capacity (57). This type of emotion-regulation deficit is a core psychological vulnerability for non-suicidal self-injury, as individuals may adopt self-injury as an immediate alternative strategy to relieve intense negative affect.

Importantly, the results revealed that social isolation partially mediated the sleep disorder–non-suicidal self-injury link, with the indirect effect accounting for 38.99% of the total effect. This provides empirical support for a statistical mediation pattern linking sleep disorder-social isolation-non-suicidal self-injury, which can be interpreted from two angles. First, sleep disorder may be related to social isolation through impairments in social functioning: daytime fatigue, attentional problems, and irritability can reduce adolescents' willingness and ability to engage in peer activities and may also undermine parent–child communication, gradually increasing perceived psychological distance from others. For adolescents with allergic rhinitis, recurrent symptoms already constrain social participation; when combined with sleep problems, weakened social connectedness may become even more pronounced. Second, the association between social isolation and non-suicidal self-injury aligns with interpersonal theories, which emphasize thwarted belongingness as a central driver of self-injury and suicide-related behaviors (58). When adolescents experience strong alienation, unmet belonging needs may trigger helplessness, loneliness, and negative self-evaluations (59). In the absence of effective support, non-suicidal self-injury—private, immediate, and negatively reinforcing—may be selected to temporarily escape or regulate psychological pain.

Notably, sleep disorder remained significantly associated with non-suicidal self-injury after inclusion of social isolation in the model, suggesting that additional factors may also be involved in this association beyond social isolation. Potential mechanisms include reduced impulse control, nightmare-related or nocturnal awakening–related activation of distressing experiences, and cognitive impairment due to chronic fatigue. These alternative pathways warrant further investigation.

### 4.2 Person-centered findings

Using latent profile analysis, this study identified two qualitatively distinct subgroups: a “low sleep disorder–low social isolation” group and a “high sleep disorder–high social isolation” group. This classification demonstrates meaningful heterogeneity in sleep and psychosocial functioning among adolescents with allergic rhinitis, addressing a limitation of variable-centered approaches that may obscure subgroup differences.

The high sleep disorder–high social isolation subgroup comprised 53.1% of the sample, suggesting that more than half of adolescents with allergic rhinitis face a dual burden of impaired sleep and weakened social connectedness. This pattern resonates with conservation of resources theory, which posits that resource loss can be cascading: sleep problems may be related to reduced energy and social interaction capacity, which could be accompanied by greater interpersonal withdrawal; similarly, lower perceived social support may coincide with poorer coping with illness stress and less favorable sleep routines—forming a vicious cycle of “sleep disorder → social isolation → further sleep deterioration.” In contrast, the low sleep disorder–low social isolation group, despite having allergic rhinitis, maintained relatively favorable sleep and social functioning, possibly reflecting protective factors such as lower disease severity, stronger coping resources, or better support systems.

The significant between-profile difference in non-suicidal self-injury further supports the relevance of co-occurring risk factors. Adolescents in the high sleep disorder–high social isolation group reported higher non-suicidal self-injury than those in the low-risk adaptive group, consistent with the cumulative risk model (60), where multiple risks jointly confer greater vulnerability than any single risk alone. Clinically, adolescents presenting both severe sleep problems and high social isolation should be considered a priority high-risk subgroup for non-suicidal self-injury screening and intervention.

### 4.3 Theoretical and practical implications

This study offers three main theoretical contributions. First, it extends research on the sleep disorder–non-suicidal self-injury association from general adolescents to a chronic physical illness population, enriching understanding of psychological-behavioral mechanisms under somatic disease conditions. Prior allergic rhinitis research has largely focused on pathophysiology and pharmacotherapy, often overlooking psychosocial consequences; the present findings suggest allergic rhinitis may be not only a physical condition but also a meaningful risk context for adolescent mental health problems, supporting interdisciplinary integration between otolaryngology and mental health services. Second, by introducing social isolation as a mediator, this study clarifies a plausible psychological pathway linking sleep problems to non-suicidal self-injury and broadens the application of interpersonal theories in adolescents with chronic illness. Social isolation, as an indicator of compromised belongingness, appears to serve as a bridge connecting symptom burden to self-injurious behavior, highlighting a potential intervention target. Third, the combined use of variable-centered and person-centered approaches provides both overall association patterns and subgroup heterogeneity, strengthening robustness and potential generalizability of the conclusions.

In managing adolescents with allergic rhinitis, clinicians should incorporate routine sleep assessment, providing sleep hygiene guidance and/or referral to sleep medicine when needed. Clinicians should be alert to social functioning impairment, particularly among those reporting high social isolation, who may benefit from psychological counseling or social work services aimed at strengthening connectedness and belonging. For the identified high-risk subgroup (high sleep disorder + high social isolation), integrated interventions targeting both sleep and psychosocial difficulties are recommended. School-based mental health programs may include adolescents with chronic illnesses such as allergic rhinitis as a focus group, using peer support and group interventions to reduce alienation and thereby help prevent non-suicidal self-injury.

### 4.4 Limitations and directions for future research

Several limitations should be noted. First, the cross-sectional design precludes causal inference. Sleep disorder, social isolation, and non-suicidal self-injury may be bidirectionally or cyclically related; for example, non-suicidal self-injury may worsen sleep and social functioning through guilt, shame, or heightened distress. Future studies should use longitudinal designs with repeated measurements to clarify temporal ordering and causality.

Second, although participants were recruited from five tertiary hospitals, the sample was derived from a hospital-based consecutive sampling process rather than a strict probability random sample. Therefore, the findings should be interpreted with caution in terms of representativeness and generalizability. Adolescents who sought care at tertiary hospitals may differ from those in community settings or primary care facilities in symptom severity, healthcare access, or psychosocial characteristics. Future studies should employ probability-based or school-/community-based sampling strategies across multiple regions to improve representativeness.

Third, all variables were self-reported, which may introduce social desirability and recall bias. Although Harman's test suggested common method bias was not severe, future work could incorporate objective sleep measures (e.g., actigraphy or polysomnography) to enhance measurement validity.

Fourth, the study tested social isolation as a single mediator, but multiple mechanisms may link sleep disorder to non-suicidal self-injury. Future research could examine additional mediators (e.g., emotion regulation difficulties, rumination, impulsivity, depressive symptoms) and potential moderators (e.g., family functioning, peer relationship quality, personality traits) to build a more comprehensive model.

Another important limitation concerns the medication-related exclusion criterion. To reduce pharmacological confounding in the assessment of sleep disturbance, we excluded adolescents who had used medications within 2 weeks prior to data collection that might affect sleep architecture or subjective sleep experience, including antihistamines and intranasal corticosteroids. Although this approach may have improved internal validity by reducing medication-related influences on sleep measurement, it may also have compromised the representativeness of the sample, because these medications are part of standard treatment for allergic rhinitis in routine clinical practice. As a result, the study sample may underrepresent adolescents receiving standard care and may be more reflective of untreated, undertreated, or temporarily unmedicated patients. This may limit the generalizability of the findings to the broader population of adolescents with allergic rhinitis. In addition, because medication use may be associated with disease severity, healthcare-seeking behavior, treatment adherence, and symptom control, this exclusion criterion may have introduced selection bias and may have influenced the observed magnitude of associations among sleep disorder, social isolation, and non-suicidal self-injury. Future studies should include medicated patients and treat medication exposure as a covariate, stratification factor, or moderator in order to better reflect real-world clinical settings.

Finally, the limitation is that depressive symptoms were not directly assessed and therefore could not be controlled for in the present analyses. This is important because depressive symptoms are strongly associated with sleep disturbance, social isolation, and non-suicidal self-injury in adolescents, and may have acted as a confounding factor in the observed relationships. Although participants with major psychiatric disorders were excluded, subclinical or undiagnosed depressive symptoms may still have been present. Accordingly, the associations reported in this study should be interpreted with caution, and future research should incorporate validated measures of depressive symptoms to determine whether these relationships remain after adjustment for affective symptom burden.

## 5 Conclusion

Using a combined variable-centered and person-centered approach in adolescents with allergic rhinitis, this study systematically examined the association between sleep disorder and non-suicidal self-injury and the potential role of social isolation. The results showed that sleep disorder was significantly associated with non-suicidal self-injury, and this association was partially statistically mediated by social isolation. Latent profile analysis further identified two distinct subgroups, with the high sleep disorder–high social isolation group showing higher non-suicidal self-injury than the low-risk adaptive group. These findings extend current understanding by highlighting possible explanatory patterns and heterogeneity among adolescents with allergic rhinitis, and they provide empirical support for risk stratification and targeted assessment in clinical practice. However, longitudinal studies are needed to clarify temporal ordering and causal relationships.

## Data Availability

The raw data supporting the conclusions of this article will be made available by the authors, without undue reservation.
